# Evaluation of the virucidal efficacy of disinfectant wipes with a test method simulating practical conditions

**DOI:** 10.1186/s13756-019-0569-4

**Published:** 2019-07-16

**Authors:** Britta Becker, Lars Henningsen, Dajana Paulmann, Birte Bischoff, Daniel Todt, Eike Steinmann, Joerg Steinmann, Florian H. H. Brill, Jochen Steinmann

**Affiliations:** 1 Dr. Brill + Partner GmbH Institute for Hygiene and Microbiology, Norderoog 2, 28259 Bremen, Germany; 2 Faculty of Medicine, Department for Molecular and Medical Virology, Ruhr University Bochum, Bochum, Germany; 3 Institute of Hospital Hygiene, Medical Microbiology and Clinical Infectiology, Paracelsus Medical University, Nuremberg, Germany

**Keywords:** Virucidal efficacy, Wipes, 4-field test, Virus transfer, Surfaces

## Abstract

**Background:**

The use of disinfectant wipes in hospitals is increasing over the last years. These wipes should be able to inactivate microorganisms including viruses on environmental surfaces and to prevent their transfer to clean areas.

The European norm (EN) 16615:2015 describes a wiping process over four fields starting on the contaminated field 1 followed by fields 2–4 and back to the starting point (4-field test). This test method exclusively describes killing and transfer of vegetative bacteria and fungi by disinfectant wipes without measuring virucidal activities. Therefore, it was the aim of this study to use the existing test methodology additionally to evaluate virus inactivation by wipes.

**Methods:**

The 4-field test was performed with four commercially available disinfectant wipes including the examination of the active solutions of these wipes with a reference wipe. Murine norovirus (MNV) as surrogate of human noroviruses, adenovirus (AdV) type 5 and polyomavirus SV40 (SV40) were chosen as test viruses.

**Results:**

The per acetic acid (PAA)-based wipe (wipe A) was able to inactivate all three test viruses resulting in a four log_10_ reduction on test field 1, whereas the quaternary ammonium compound (QAC)-based products (wipes B and C) failed to reach such reduction. Both QAC-based wipes were able to inactivate SV40 and only the active solution of wipe B was effective against MNV. Another wipe with 2-propanol as active ingredient (wipe D) was not able to show a sufficient efficacy against all three test viruses. There was a good agreement between the results of the wipes and the corresponding fluids showing no influence of the material of wipes.

Tests with the 2-propanol-based wipe D showed a transfer of all test viruses to the non-contaminated test fields 2–4. SV40 was additionally transferred by the QAC-based wipe C with 0.78% active ingredients to these additional fields. In all other cases no virus transfer to test fields 2–4 was observed. Finally, no virus could be detected in the PAA-based wipe A after usage in the 4-field test in contrast to the other wipes examined.

**Conclusions:**

The successful performance of a 4-field test with viruses demonstrated that the existing wiping method with bacteria and fungi can be used in addition for measuring virucidal efficacy. The virus-inactivating properties of surface disinfectants could be evaluated therefore with a test simulating practical conditions with mechanical action resulting in more reliable data than the existing quantitative suspension tests and/or a carrier test without any mechanical action.

## Background

Microorganisms like gram-positive and gram-negative bacteria can persist on inanimate surfaces for prolonged time [1]. In addition, Kramer and co-workers described that viruses can persist for few hours up to months [1]. Furthermore, it was noted that in general the group of non-enveloped viruses are more stable on environmental surfaces than the enveloped ones [1].

Surfaces can become contaminated by hands, objects, settling of virus containing aerosols or contaminated fluids [2]. Therefore, these surfaces may play an important role for transmission of pathogens in the hospital [3, 4]. In contrast, the detection of viruses on environmental surfaces in the hospitals is rarely reported. Gallimore et al. described the detection of mainly norovirus and rotavirus in environmental swabs from two pediatric wards [5]. In an adult intensive care unit in Brazil group A rotavirus (RV-A) was detected by reverse-transcriptase polymerase chain reaction (PCR) in environmental surface samples. Here RV-A was regarded as biomarkers for viral contamination [6]. In addition, astroviruses were found in a pediatric primary immunodeficiency unit [7]. In a pediatric outpatient waiting area most frequently adenovirus was detected in environmental samples [8]. In summary, it was estimated that more than 30% of all hospital-acquired infections in many pediatric settings may be caused by viruses [2].

Meanwhile, contaminated surfaces like “high-touch” environmental surfaces (HITES) in critical areas of the hospitals were identified [9] and these HITES may be also responsible as vehicles for human pathogenic viruses. Therefore, careful cleaning and disinfection of environmental surfaces in hospitals and medical wards is an important step in infection control and part of many prevention programs in healthcare. The methods are based on a wide range of technologies including liquid disinfectants, self-disinfecting surfaces like copper or silver and the vaporisation of peroxides and other chemicals [10].

Disinfection of surfaces by manually performed wiping as one important part in the healthcare setting increased in the last years. Recently, it has been shown that a pre-impregnated wipe with sporicidal efficacy demonstrated superiority in comparison to a cloth soaked in 1,000 ppm chlorine solution underlining the increasing importance of disinfectant wipes [11].

For disinfectant wipes, the virucidal testing often starts with a quantitative suspension test with the soaking solution or the squeezed-out liquid followed by a test simulating practical conditions. However, there is no European Norm (EN) measuring the virus-inactivating properties by wiping.

After intensive work by the group of J. Gebel in Bonn, Germany a “4-field test” was developed which is now the EN 16615:2015 [12]. Here, the ability of disinfectant wipes to remove bacteria and fungi from a contaminated test field 1 and the potential transfer between clean surfaces (test fields 2–4) can be measured. Until now, this method was only described with different bacteria and *Candida albicans*. In parallel, a test of hygienic towelette wipe efficiency was developed for bacteria with the Wiperator (Filtaflex ltd, Almonte, Ontario, Canada, http://www.filtaflex.ca/wiperator.htm), which is now the basis of the ASTM E2967–15 [13]. Meanwhile, this ASTM method was evaluated carefully in three independent laboratories [14]. Here, two species of vegetative bacteria, one gram-positive coccus (*Staphylococcus aureus)* and one gram-negative bacillus (*Acinetobacter baumannii)* were chosen as microorganisms but again no viruses. In addition, data with both vegetative bacteria and *Clostridioides difficile* spores have been recently published using detergent wipes [15].

Importantly, until now no data with viruses have been generated with both methods described above. Therefore, it is still unclear whether the existing 4-field test method or the Wiperator technology can be transferred to test methods using viruses. Finally, an appropriate virus-inactivating claim should be possible and should help to prevent nosocomial virus infections.

We used the European test method described for bacteria EN 16615:2015 [12] by incorporating important non-enveloped viruses and started to develop a test for wipes measuring inactivation and transfer to clean areas in one manual step. The choice of viruses was influenced by the existing test viruses from in vitro tests like the EN 14476:2015 [16] and the German Guideline of Deutsche Vereinigung zur Bekämpfung der Viruskrankheiten e.V. (DVV) and Robert Koch-Institute (RKI) [17].

## Materials and methods

### Test viruses

The murine norovirus S99 (MNV) was obtained from Dr. E. Schreier, former head of FG15 Molecular Epidemiology of Viral Pathogens at the RKI in D-13302 Berlin. The adenovirus (AdV) type 5 strain adenoid 75 originated from PD Dr. A. Heim, Institute of Medical Virology, Hannover Medical School, D-30625 Hannover and the polyomavirus SV40 strain 777 (SV40) was obtained from PD Dr. A. Sauerbrei, Institute of Virology and Antiviral Chemotherapy at the Friedrich Schiller University of Jena, D-07747 Jena.

### Virus propagation

The test virus suspensions were prepared by infecting monolayers of the respective cell lines. The virus titres of these suspensions ranged from 10^6^ to 10^9^ TCID_50_/ml. MNV was propagated in RAW 264.7 cells (a macrophage-like, Abelson leukemia virus transformed cell line derived from BALB/c mice, ATCC TIB-71) and adenovirus in A549 cells (human lung epithelial carcinoma cells) which originated from the Institute of Medical Virology, Hannover Medical School. Polyomavirus SV40 strain 777 was propagated in CV-1 cells (kidney cells of African green monkey, Friedrich-Loeffler-Institute RIE 185). The Minimum Essential Medium was supplied by Lonza Verviers, Belgium and the fetal calf serum (FCS) by Biochrom GmbH, Germany.

### Wipes

Four commercial disinfectant wipes were examined in the 4-field test. Wipe A is a disinfectant wipe based on 0.06% per acetic acid with a bactericidal, virucidal and sporicidal claim. Wipe B is based on 0.6% quaternary ammonium compounds (QACs) with a claim against bacteria, spores and viruses. The active ingredients of wipe C are 0.78% QACs with a claim against bacteria, enveloped viruses, norovirus and SV40. Wipe D is based on 70% (v/v) 2-propanol with a use mainly in clean rooms and with a claim against bacteria only.

In addition, the active solutions of all wipes were examined in combination with a reference wipe. The reference wipe was the Tork Standard wipe, art. no. 90491 supplied by SCA Tork (D-68305 Mannheim) (17.5 cm × 28 cm, 55% pulp, 45% polyethylenterephthalat) as described in the EN 16615:2015 [12]. All experiments were performed in two independent runs.

### 4-field test with viruses

**Fig. 1:**
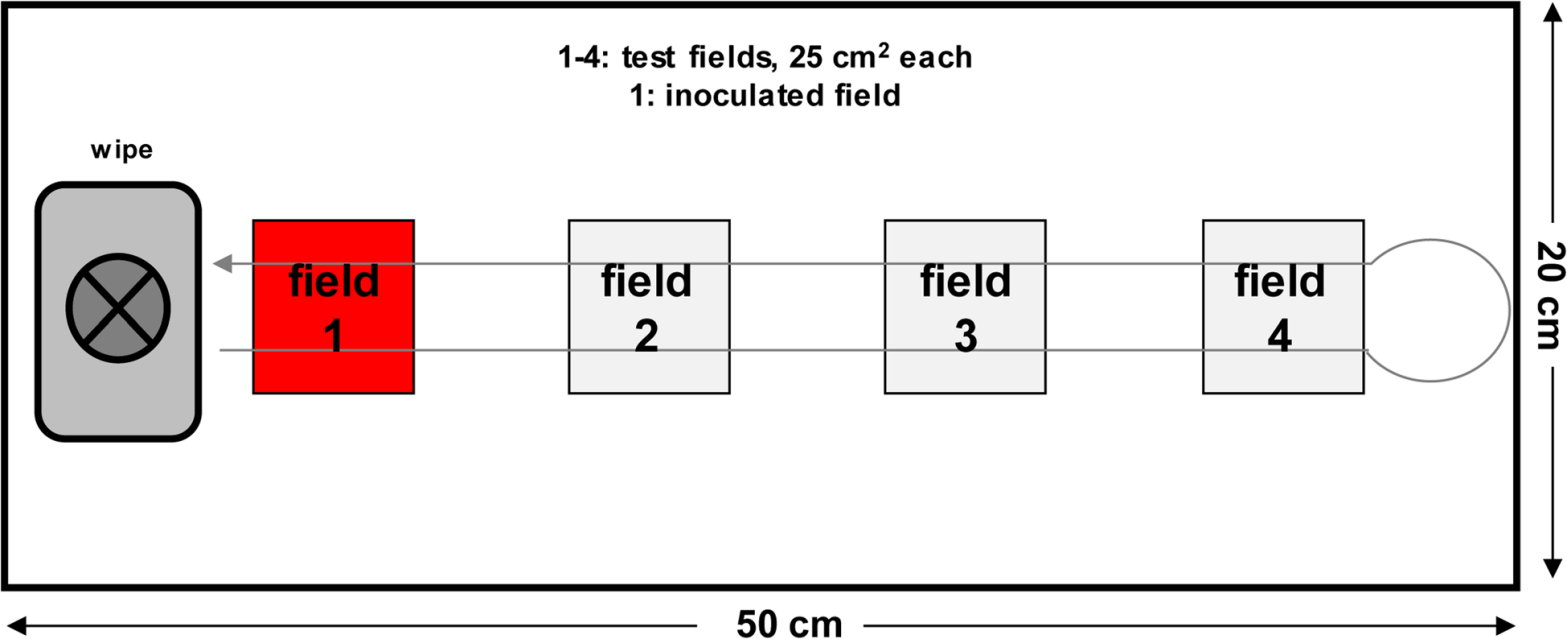
PVC material with polyurethane surface coating measuring 20 × 50 cm is prepared by recording four 5 × 5 cm squares. Test field 1 is contaminated with a defined amount of virus inoculum (virus suspension + interfering substance). This is followed by a wiping process (2-s motion) with a wipe, which was fixed under a unitary weight. After a defined exposure time of the test substance, the remaining test viruses are recovered from all four test fields with swabs. The aim is to show a reduction of the test viruses on test field 1 and the carryover to the previously virus-free test fields 2–4 (schematic drawing according to European Standard EN 16615:2015)

The performance of the 4-field test is described in detail in the EN 16615:2015 as European Standard for measuring of efficacy of disinfectant wipes against bacteria and *C. albicans* [12]. Briefly, four squares as test fields were marked on a PVC with PUR surface coating material (20 cm × 50 cm), figuring a row at a distance of 7 cm from one another (Fig. 1). The marked test field 1 on this flooring was inoculated with the inoculum based on the test virus suspension and a solution of interfering substance (clean conditions, 0.3 g/L BSA). Here, 50 μl inoculum was pipetted on the first test field (field 1) and distributed with a glass spatula. Immediately after drying of the inoculum on test field 1 at 20 °C – 25 °C the wipe was fixed under a unitary weight (granite block with a weight between 2.3–2.5 kg). This unitary weight should simulate the average pressure during the wiping process. For the examination the granite block with the fixed wipe was rapidly moved from test field 1 to test field 4 and back within no longer than 2 s. At the end of the contact time (5 min chosen for all experiments) the test organisms were recovered from all four fields with a moistened and a dry nylon swab (FloQSwab, art. no. 529CS0, Copan Diagnostics Inc., Mantua, Italy) as described in the EN 16615:2015 [12]. Swabs of each field were transferred to 5 ml Minimum Essential Medium (MEM), respectively and tubes were vortexed for 60 s. Virus titres of the eluates were determined immediately by end point dilution techniques as described in the EN 14476:2015 [16] and calculated using the method of Kärber [18] and Spearman [19]. The virus titre is expressed as log_10_TCID_50_/ml with 95% confidence interval. The virus reduction was calculated by comparing the virus titres of each test field with those immediately after drying and the chosen exposure time.

### Controls in the 4-field test with viruses

The following controls were included:

#### Initial virus control (VIC)

For calculating the initial virus titre 0.05 ml inoculum was mixed with 5 ml MEM followed by determination of the virus titre by end point dilution titration on permissive cells.

#### Drying control

Drying controls were performed immediately after drying of the virus inoculum on a test field (DCt0) and after the defined exposure time of 5 min (DCt5) with the same recovery procedure as described above. The DCt0 shows the loss of virus during the drying process. DCt5 was the reference for calculation of the reduction factor (RF) on the test fields 1–4, respectively.

#### Water control

To discriminate the virus-inactivation properties of the wipes from a mechanical effect on test field 1 two water controls (water of standardized hardness (WSH) and Aqua bidest.) with the Tork Standard wipe were included. In addition, the virus titres were measured on test fields 2–4 for control of the virus transfer with water.

#### Cytotoxicity control

Test field 1 was inoculated with an inoculum with MEM instead of virus suspension. A run with the respective wipe and the elution followed. Finally, the eluate of test field 1 was added to the corresponding cell cultures. This cytotoxicity control defines the lower detection limit of the test system for the corresponding wipe.

#### Neutralisation control (NC)

To exclude that the resulting eluate after immediate dilution is still expressing any virus-inactivating property causing false-positive results, 1.25 ml of the resulting eluate of the cytotoxicity control was contaminated with 12.5 μl test virus suspension and stored for 30 min. Finally, the virus titre was determined by end point dilution method. For test validation the difference between NC and VIC should be ≤0.5 log_10_ steps as described in the EN 14476:2015 [16].

#### Interference control (IC)

Here it must be excluded that the residual product in the eluate will influence the infectivity of the cells and thus might inhibit the virus propagation causing false-positive results. 2.5 ml of the eluate of the cytotoxicity control or phosphate-buffered saline (PBS) was mixed with 2.5 ml of a double-concentrated cell suspension and stored for 1 h at 37 °C. Afterwards the cells were re-suspended and the virus suspension was titrated with these cells. The PBS treated cell suspension served as a control. For validation of the test the difference between both assays should be lower than 1.0 log_10_ steps as described in the EN 14476:2015 [16].

### Virus detection in the wipes after usage

The wipes were examined for virus after the usage. Here, the area used for wiping was cut out and this material was transferred in a 50 ml tube with 10 ml MEM without fetal calf serum (FCS). After vortexing for 30 s and a squeezing-out step with a sterile glass spatula 100 μl of each fluid was analysed for virus by microtitration on the appropriate cell lines.

## Results

### Performance test control measures

**Fig. 2:**
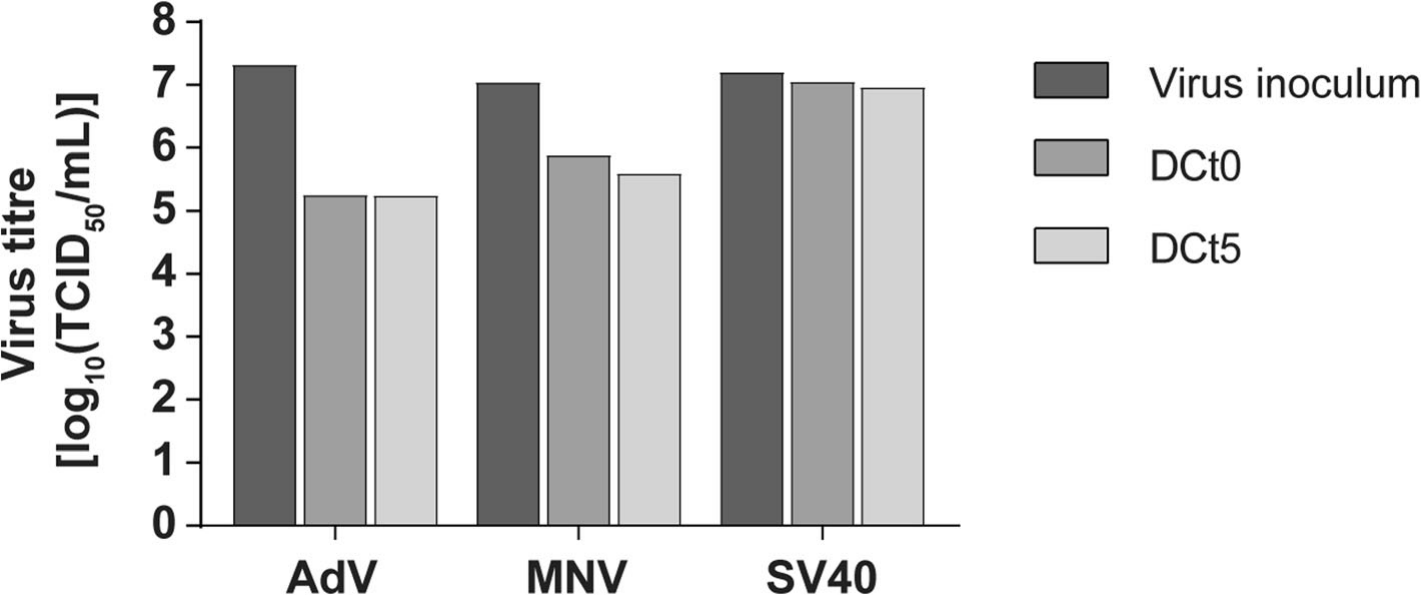
Stability of three test viruses adenovirus (AdV) type 5, murine norovirus (MNV) and polyomavirus SV40 (SV40) under clean conditions immediately after drying (DCt0) and after 5 min exposure time (DCt5) in comparison to the inoculum without drying (VIC). The calculated reductions of virus titre after 5 min were 0.34 for SV40, 1.47 for MNV and 2.04 for AdV

In the beginning, the virus titre of the inoculum (VIC) was compared with the titres on the PVC flooring immediately after being visibly dry (DCt0) and after 5 min exposure time (DCt5). Results are shown in Fig. 2. SV40 was the most stable virus (reduction 0.15 log_10_ steps after drying) followed by MNV (1.16) and AdV (2.07). The additional exposure time of 5 min only produced minor changes of the virus reduction for all three test viruses.

**Fig. 3:**
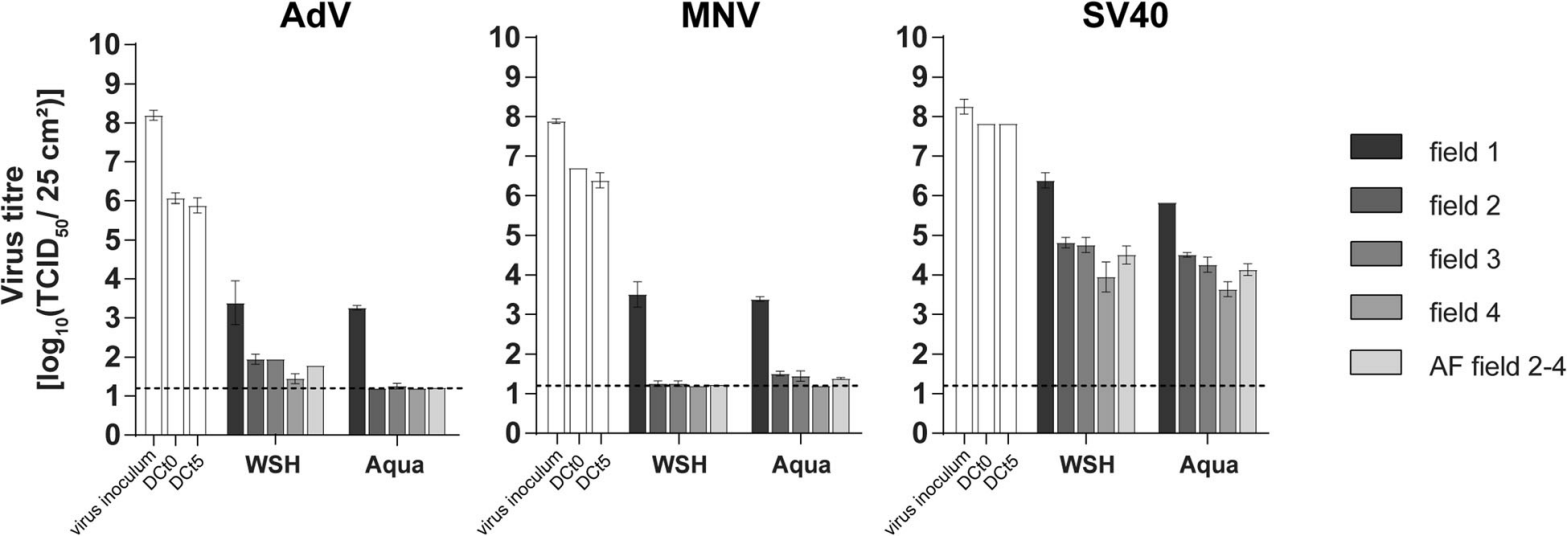
The Tork Standard wipes were treated with water of standardized hardness (WSH) and Aqua bidest. (Aqua) for studying the mechanical removal from test field 1 and the transfer to test fields 2–4 with AdV, MNV and SV40. The titres of the inoculum, after drying (DCt0) and 5 min exposure time (DCt5) together with titres on test fields 1–4 and a summary of transfer to test fields 2–4 (accumulation factor (AF) fields 2–4) are shown. The lower detection limit defined by the cytotoxicity is indicated by a dashed line

With the water control the transfer of the dried virus from test field 1 to the other test fields is visible. The reference wipe Tork Premium Spezial Tuch was treated with water of standardized hardness (WSH) and with Aqua bidest. in parallel. After wiping and the chosen exposure time, virus titres on test fields 1–4 of the water controls were detected. Additionally, virus titres of DCt0 and DCt5 were measured (Fig. 3).

Examining MNV the calculated loss of virus titre after the drying step and water treatment causes RFs of 2.88 (WSH) and 3.00 (Aqua bidest.). This virus loss was the highest among the three test viruses followed by AdV. Here the RFs were 2.50 (WSH) and 2.63 (Aqua bidest.). When testing SV40 only a small reduction of virus titre was observed after drying and water treatment (RFs = 1.44 with WSH and 2.00 with Aqua bidest). In contrast, the highest titres on the other test fields were observed with SV40 demonstrating a great transfer of this virus to test fields 2–4. With AdV and MNV only lower virus titres were measured on test fields 2–4 (Fig. 3). In the EN 16615:2015 it is requested to demonstrate in the water control a transfer of bacteria and *C. albicans* to the test fields 2–4 [12] which is also shown here with the test viruses. Concerning removal from test field 1 and transfer to test fields 2–4 resulting data with WSH and Aqua bidest. were nearly identical.

### Virucidal efficacy of the wipes

**Fig. 4:**
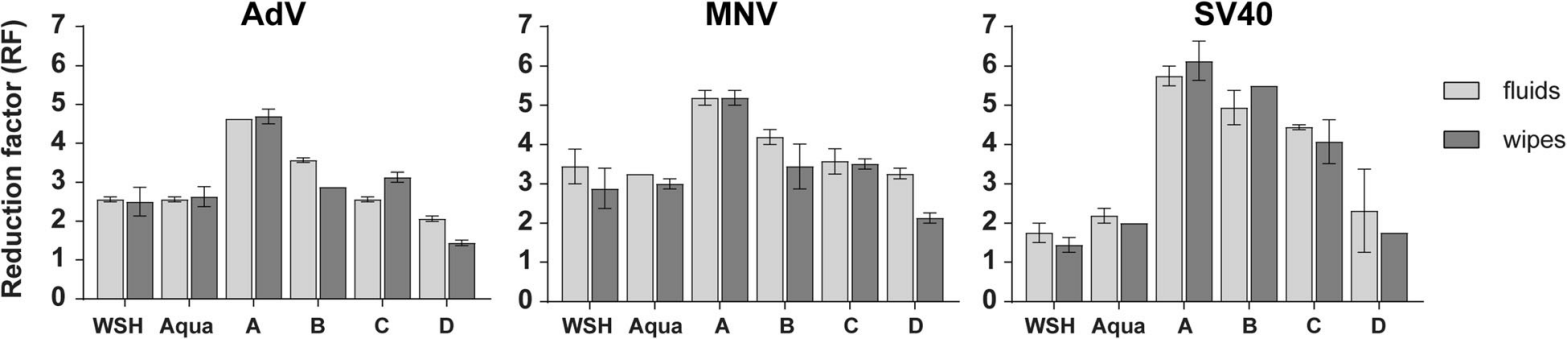
Virucidal properties (reduction factor on field 1) of four commercial wipes and the corresponding fluids in comparison with WSH and Aqua bidest. Against AdV, MNV and SV40 in the 4-field test. The left columns (light grey) show the efficacy of the fluids with the Tork Standard wipe and the right columns (dark grey) of the commercial disinfectant wipes by giving the reduction factor (RF). A four log_10_ reduction (inactivation 99.99%) is considered for efficacy

The four wipes exhibited different virucidal efficacies against the chosen viruses (Fig. 4). The PAA-based wipe (wipe A) was able to achieve a four log_10_ reduction on test field 1 against all three test viruses. In contrast, wipe B and C were not able to inactivate sufficiently MNV and AdV on test field 1. However both QAC-based wipes were active against SV40 (≥ four log_10_ steps on test field 1). The 2-propanol-based wipe (wipe D) was not able in inactivate any of the three test viruses to the desired extent.

The results with the pre-wetted wipes and the active fluids tested with the Standard Tork wipe were nearly identical (Fig. 4). Only the results of wipe B and the corresponding active solution were different. The active solution of wipe B (QAC-based formulation) was effective against MNV resulting in a four log_10_ reduction (RF = 4.19), whereas the corresponding wipe failed (RF = 3.44) thus producing a nearly identical RF than WSH (RF = 2.88) and Aqua dest. (RF = 3.00).

A remarkable transfer to fields 2–4 was only seen when testing the 2-propanol-based wipe D with all three viruses (Table 1). Additionally, SV40 was transferred to test fields 2–4 by the product C. In all other experiments no transfer of viruses from test field 1 to the other fields was measured (Table 1).

**Table 1:** Transfer of the three test viruses AdV, MNV and SV40 to fields 2, 3 and 4 when using the four different wipes. The virus titres are given as TCID_50_/ml on test fields 2–4 (CT = cytotoxicity, no virus = no virus detected)

	AdV			MNV			SV40		
	field 2	field 3	field 4	field 2	field 3	field 4	field 2	field 3	field 4
Aqua	1.33 ± 0.13	1.33 ± 0.13	1.23 ± 0.04	1.39 ± 0.07	1.33 ± 0.07	1.20 ± 0.00	4.42 ± 0.10	4.11 ± 0.10	3.70 ± 0.19
Wipe A	no virus	no virus	no virus	no virus	no virus	no virus	no virus	no virus	no virus
Wipe B	no virus	no virus	no virus	no virus	no virus	no virus	no virus	no virus	no virus
Wipe C	no virus	no virus	no virus	no virus	no virus	no virus	3.45 ± 0.25	3.32 ± 0.13	3.76 ± 0.07
Wipe D	1.51 ± 0.06	1.51 ± 0.19	1.57 ± 0.38	1.76 ± 0.44	1.51 ± 0.06	1.39 ± 0.06	4.70 ± 0.13	4.95 ± 0.13	4.57 ± 0.38

### Examination of the wipes for viral contamination

**Fig. 5:**
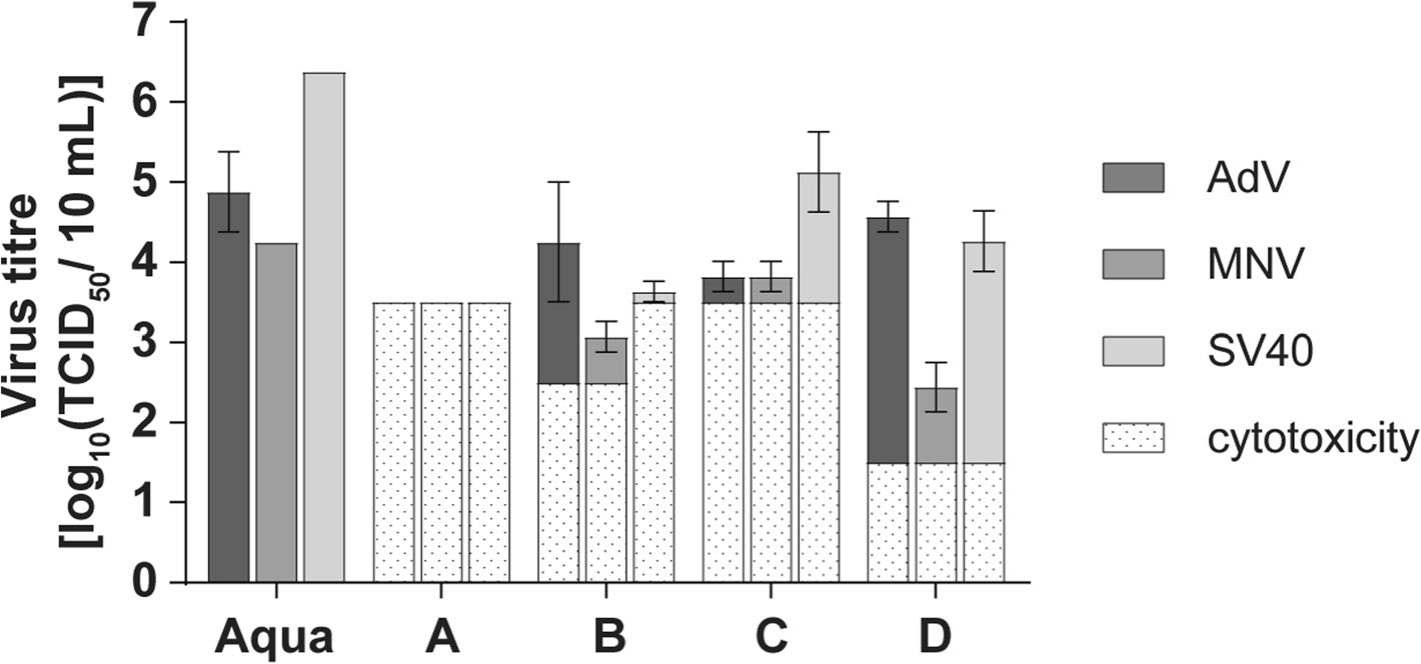
Determination of residual virus in the four wipes (**a**-**d**). Virus titres are given as log_10_ TCID_50_ in 10 ml. No residual virus was detectable in the PAA-based wipe (wipe A). The lower detection limit is here defined by the cytotoxicity

Next, the wipes were examined for contamination with viral material. Figure 5 shows that in the PAA-based wipe A no residual virus could be detected after usage. In all other wipes and in the Standard wipe treated with Aqua bidest. MNV, AdV and SV40 could be detected (Fig. 5).

## Discussion

Virus transfer in the hospital can be interrupted by the appropriate cleaning and disinfection of surfaces. Pre-wetted wipes may play a role in this [20]. Meanwhile, detergent and disinfectant wipes with a proven efficacy against bacteria and *C. albicans* are available based on data of the 4-field test described in the EN 16615:2015 [12] or the ASTM E2967–15 [13].

The virucidal claim of these pre-wetted wipes in Europe is nowadays nearly completely based on quantitative suspension tests only like the EN 14476:2015 [16] or the German Guideline of DVV and RKI [17]. In some cases a test simulating practical conditions without mechanical action is performed in addition [21]. However, a claim against viruses with a practical test like the 4-field test with mechanical action including inactivation and removal steps would provide more precise information for these pre-wetted wipes used in healthcare.

Meanwhile, there are two standards designed to measure the claims of disinfectant pre-wetted wipes. We have chosen the EN 16615:2015 [12] in contrast to the ASTM E2967–15 [13] with the Wiperator due to the possibility to check the transfer of bioburden in one process and the shorter wiping procedure. The EN 16615:2015 describes a manual horizontal movement (2 s) in contrast to the orbital mechanical rotation (10 s) with the Wiperator. The possible transfer in the EN 16615:2015 can be checked when examining the virus load on test fields 2–4. In contrast, only when introducing an additional step the Wiperator provides information on the bacterial transfer from the wipe to three consecutive stainless disks together with the effect of the mechanical action (10 s, 150 g pressure) [22].

Our choice of the test virus was mainly influenced by existing suspension tests [16, 17]. In addition, AdV and MNV as a surrogate of human norovirus are also test viruses in the prEN 16777:2016 [21] and the German Guideline of DVV [23]. SV40 is a test virus in the German Guideline of DVV/RKI for testing disinfectants in suspension and was introduced in the past as a surrogate of polyomaviruses [17]. Therefore, tests with mechanical action should include viruses that have been studied in quantitative suspension tests and/or in carrier tests without mechanical action.

First of all the viral stability of the three test viruses was examined after drying. SV40 was more stable during this process than MNV and AdV. The greatest decline of virus titre was found with AdV during the drying studies. However, in summary, despite the drop in virus titre with all chosen test viruses it is still possible to demonstrate a four log_10_ reduction later in the tests with the wipes necessary for claiming a sufficient efficacy. In contrast to bacteria testing four log_10_ reduction is necessary in virus testing as described in the DIN EN 14476:2015 [16] due to the fact that high titers in many cases are difficult to reach and that high cytotoxicity of the disinfectants will increase the lower detection limit.

Tests with WSH and Aqua bidest showed that the loss of virus titre on test field 1 was the greatest with MNV followed by AdV and SV40. With SV40 there was only a small loss by drying but a great virus transfer to the other test fields whereas with MNV and AdV only a small transfer resulted. This means when using wipes the transfer to consecutive surfaces might be influenced by the kind of virus contamination.

Testing the different wipes the greatest reduction of virus titre was measured with the PAA-based wipe A resulting in greater than four log_10_ steps against all three test viruses. In general, a four log_10_ reduction of titre in virus tests is necessary for claiming efficacy as in other virucidal test methods. Despite the fact, that wipe B, unlike the active solution, just missed a four log_10_ reduction, nearly identical results were found when testing the pre-wetted wipes and the corresponding fluids with the Tork Standard wipe. This means that the material of the wipes used in this study seemed to have no great impact on efficacy. Identical results were found by Wesgate and co-workers when examining different products with microfiber, cotton or non-woven materials [24]. These examinations were performed with *Pseudomonas aeruginosa* and *S. aureus* and different disinfectant solutions by the ASTM method and the EN 16615:2015.

The QAC-based wipes B and C were also able to reach efficacy four log_10_ reduction against SV40 but failed to inactivate MNV and AdV. The 2-propanol based product (wipe D) was inactive against all three chosen test viruses. These marked differences in efficacy of wipes should also be observed when testing with bacteria [15]. However in a study examining detergent wipes with *S. aureus*, *A. baumannii* and *C. difficile* all seven detergent wipes were not able to produce a sufficient reduction and all wipes transferred significant amounts of bacteria and spores to consecutive surfaces [15]. In our study, the disinfectant wipes under examination were mainly chosen with respect to their ability to already inactivate microorganisms including different viruses in suspension assays. By doing so, a virucidal activity in the 4-field test was at least expected for disinfectant wipes in comparison to the detergent wipes examined with vegetative bacteria in the other study [15].

The great efficacy of the PAA-based wipe A in the 4-field test was finally confirmed by the examination of the wipes after usage. None the three test viruses could be detected in this wipe in contrast to the others.

## Conclusions

In summary, we showed that the principle of the existing EN 16615:2015 can be transferred to the examination with viruses. Our data demonstrate that a successful virus inactivation and a prevention of virus transfer can be reached. Consequently, a 4-field test evaluating virucidal activity of disinfectant wipes is possible and will allow more precise information for virucidal claims of wipes.

Besides the possibility to introduce wipes with a virucidal claim the appropriate handling “one site-, one direction, one use” is still of great importance for hospital hygiene. Therefore, this correct management of the wipes together with an appropriate claim will help to control viral bioburden on healthcare surfaces. In addition, future studies have to address the area of activity of the chosen disinfectant wipe in comparison to detergent wipes. Finally, the detection of all test viruses in three of four wipes tested makes a careful disposal of great importance.

## Data Availability

All data generated during this study are included in the published article.

## Abbreviations

AdV: Adenovirus
ASTM: American Society for Testing and Materials
DVV: Deutsche Vereinigung zur Bekämpfung der Viruskrankheiten e.V.
EN: European Norm
HITES: High touch environmental surfaces
MEM: Minimum Essential Medium
MNV: Murine norovirus
PAA: Per acetic acid
PBS: Buffered-saline solution
PCR: Polymerase chain reaction
QAC: Quaternary ammonium compound
RF: Reduction factor
RKI: Robert Koch-Institute
RV-A: Group A rotavirus
SV40: Polyomavirus SV40
WSH: Water of standardized hardness
